# Exploring Educators’ Perceptions and Experiences of Online Teaching to Foster Caring Profession Students’ Development of Virtual Caring Skills: Sequential Explanatory Mixed Methods Study

**DOI:** 10.2196/64548

**Published:** 2025-01-15

**Authors:** Lorelli Nowell, Sonja Johnston, Sara Dolan, Michele Jacobsen, Diane L Lorenzetti, Elizabeth Oddone Paolucci

**Affiliations:** 1 Faculty of Nursing University of Calgary Calgary, AB Canada; 2 Werklund School of Education University of Calgary Calgary, AB Canada; 3 Cumming School of Medicine University of Calgary Calgary, AB Canada

**Keywords:** health care education, virtual care, telehealth, online teaching, mixed methods study, student, teaching, virtual caring skills, cross-sectional survey, interview

## Abstract

**Background:**

Professionals in caring disciplines have been pivotal in advancing virtual care, which leverages remote technologies to deliver effective support and services from a distance. Educators in these caring professions are required to teach students the skills and competencies needed to provide high-quality and effective care. As virtual care becomes more integral, educators must equip students in these fields with both interpersonal and technological skills, bridging traditional hands-on learning with digital literacy. However, there is a gap in evidence exploring educators’ perceptions and experiences of teaching caring profession students about virtual caring skills within online environments.

**Objective:**

This study aims to better understand caring profession educators’ online teaching experiences to foster student development of virtual caring skills and competencies.

**Methods:**

We used a sequential explanatory mixed methods approach that integrated a cross-sectional survey and individual interviews with educators from caring professions to better understand caring professional educators’ online teaching experiences to foster student development of virtual caring skills and competencies. The survey’s primary objectives were to examine the various elements of existing e-learning opportunities, delve into educators’ perspectives and encounters with these opportunities, and identify the factors that either facilitated or hindered online teaching practices to support students in developing virtual caring skills and competencies. The individual interview guides were based on survey findings and a systematic review of the evidence to gain deeper insights into educators’ experiences and perspectives.

**Results:**

A total of 82 survey participants and 8 interview participants were drawn from educators in the fields of education, medicine, nursing, and social work. Various instructional methods were used to help students develop virtual caring skills, including reflections on learning, online modules, online discussion boards, demonstrations of remote care, and consultation with clients. There was a statistically significant difference between educators’ level of experience teaching online and their satisfaction with online teaching and learning technologies (*P*<.001) and between educators’ faculties (departments) and their satisfaction with online teaching and learning technologies (*P*=.001). Participants identified barriers (time constraints, underdeveloped curriculum, decreased student engagement, and limited access to virtual caring equipment and technology), facilitators (clearly defined learning objectives, technology software and support, teaching support, stakeholder engagement, and flexibility), and principles of teaching virtual caring skills in online environments (connection, interaction, compassion, empathy, care, and vulnerability).

**Conclusions:**

Our study identifies the barriers, facilitators, and principles in teaching virtual caring skills, offering practical strategies for educators in caring professions. This study contributes to the growing body of educational research on virtual caring skills by offering educator insights and suggestions for improved teaching and learning strategies in caring professions’ programs. As educational practices evolve, future research should explore how traditionally in-person educators can effectively teach virtual caring skills across diverse contexts.

## Introduction

### Background

Professionals in caring fields, including educators, physicians, nurses, and social workers, have played a crucial role in the ongoing development of virtual care where remote information technologies are used to ensure quality and effective care. The shift to virtual care has paved the way for innovative approaches to delivering care services, such as online teaching; remote health care and social services; and remote assistance for individuals, families, and communities to improve their social functioning, all from a distance. These virtual interactions demand digital literacy skills and comfort with technology, skills that traditionally may not have been intentionally integrated into formal education.

As virtual caring practices become integral to care provision, it is imperative that educators support caring profession students in acquiring the interpersonal and technological competencies necessary for providing virtual care. Traditionally, educators in caring professions relied on face-to-face lectures and seminar-style instruction with work-integrated learning placements, where students gained hands-on skills and collaborated with experienced educators and practicing health professionals in settings such as K-12 classrooms, hospitals, and counseling centers [1,2].

The shift to virtual teaching and care settings has challenged caring profession educators to incorporate alternative strategies for providing essential educational experiences to students [3-5] and placed added responsibilities on caring professionals to implement virtual care effectively in practice [2,3]. While the literature has long emphasized the need to support educators in meeting students’ requirements [6,7], this need has become even more critical with the increasing prevalence of virtual care environments [8,9].

Higher education institutions have an opportunity to re-evaluate their approach to delivering online education in caring professions and identify the essential technological competencies necessary for success in today’s virtual world. Given the significant transformation in education and care delivery, it is imperative that caring professionals possess the requisite skills and competencies to adapt and thrive in these new virtual environments. However, many caring profession educators face challenges when creating effective online learning experiences to prepare students for new virtual work environments, including limited bandwidth, the lack of technological devices, unfamiliarity with technological platforms, a lack of connection with students, and a lack of student engagement [10-13]. Learning new technologies can be cumbersome and frustrating [14], and technical issues can disrupt interactions that typically occur face-to-face [15-19]. These challenges underscore the necessity for a structured, evidence-based approach to developing and implementing educational technologies in online teaching and learning contexts to support virtual caring skill development [10,20-22].

The authors recently completed a systematic review from which they identified innovative online education initiatives that harnessed learning technologies for the education of caring professionals and demonstrated a growing emphasis on assisting students in cultivating effective virtual caring practices suitable for today’s virtual environments [23]. The systematic review [23] highlighted a pressing need for greater emphasis on assessing and training educators to immerse students in digital technologies, thus fostering the development of both interpersonal and digital skills essential for delivering virtual care. More research is needed regarding educators’ experiences and perceptions of teaching virtual caring skills.

### This Study

Adding to the limited body of literature would potentially enhance the understanding of best practices in online instruction to promote the development of virtual caring skills. Therefore, we conducted this study to answer the following research questions: (1) How do caring professions’ educators *describe* the online instructional methods used that support student development of virtual caring skills and competencies? (2) What are caring professions’ educators’ *experiences and perceptions* of online learning opportunities for helping students develop virtual caring skills and competencies? and (3) What are the *facilitators and barriers* to creating and engaging in online teaching that supports students’ development of virtual caring skills and competencies?

## Methods

### Design

We adopted a sequential explanatory mixed methods study design [24] to gather, analyze, and integrate quantitative and qualitative data. We used a cross-sectional survey and conducted individual interviews to gain insights into the online teaching experiences of educators in caring professions in supporting students to develop virtual caring skills and competencies. The integration of the 2 research phases became apparent when the design of the interview guide was informed by the survey findings, enabling us to delve deeper into the results obtained from the survey. Furthermore, integration occurred as we used the qualitative findings to better understand the quantitative findings, ultimately forming interpretations from the integrated findings.

### Sample and Participants

Voluntary participation was sought from educators in caring professions, including education, medicine, nursing, and social work (including those cross appointed to arts and veterinary medicine) across a midsized research-intensive institution in western Canada. Any self-reported educators from the abovementioned faculties were included in the study. No completed surveys or interviews were excluded.

### Data Collection

We crafted a survey using established methods as outlined by Rattray and Jones [25]. The survey’s primary objectives were to examine the various elements of existing e-learning opportunities, delve into educators’ perspectives and encounters with these opportunities, and identify the factors that either facilitated or hindered online teaching practices to support students in developing virtual caring skills and competencies. The survey encompassed a combination of Likert scale, closed-ended, and open-ended questions, covering demographics, experiences, instructional methods, satisfaction levels, technology use, effectiveness, and readiness. To ensure the survey’s validity, both in terms of face and content, we conducted a pilot study with a sample of 10 educators who did not participate in the study. Their suggested edits were incorporated into the survey before its dissemination.

To distribute the survey securely, we used an online platform, Qualtrics (Qualtrics International Inc). Our recruitment efforts spanned various channels such as email, Twitter (subsequently rebranded as X), Instagram (Meta Platforms), and Facebook (Meta Platforms), mirroring the methods used in prior studies [26,27]. Completion of the survey was considered as an indication of informed consent. In addition, we invited all survey participants to share their email addresses if they were interested in participating in a follow-up interview.

To gain deeper insights into educators’ experiences and perspectives, we developed a semistructured interview guide based on the findings from a systematic review [23] and the responses received in the survey. We reached out to all survey participants who provided their email addresses and conducted interviews lasting between 30 and 60 minutes via the Zoom (Zoom Communications) platform. Before each interview, we confirmed oral consent, and the sessions were audio-recorded and transcribed verbatim.

### Data Analysis

The closed-ended survey responses were obtained from Qualtrics and subsequently imported into the SPSS (version 28; IBM Corp) statistical software package for analysis. Descriptive statistics were calculated to summarize the characteristics of the study sample, including factors such as age, gender, faculty affiliation, length of time in current position, and previous experience with online teaching and learning technologies. Variations in data distribution were summarized and visually presented through tables and graphical representations, following the guidelines outlined by Polit and Beck [28]. In addition, 1-way ANOVA and Kruskal-Wallis *H* tests were conducted to analyze differences in satisfaction and likelihood to use online teaching and learning technologies in the future to support students in developing virtual caring skills. These analyses were conducted as deemed appropriate, following the recommendations of Polit and Beck [28]. To enhance readability and facilitate subsequent post hoc analyses, participant-reported ages were collapsed into 4 categories: ≤39, 40-49, 60-59, and ≥60 years. Team members with experience in statistical analysis met and contributed to ensure the accuracy of these findings.

For the analysis of open-ended survey responses and interview transcripts, each was assigned a unique identifier and imported into NVivo (version 14; Lumivero) to manage qualitative data. Our qualitative data analysis followed a thematic approach using an inductive process, aligning with the methods proposed by Braun and Clarke [29] and Nowell et al [30]. To gain a comprehensive understanding of the data, 2 researchers (LN and SJ) independently reviewed the entire qualitative dataset. Consensus coding was completed as both researchers coded the same transcripts and compared results on a one-to-one basis. Each researcher assigned sections of text to relevant codes, and the coding was then merged and discussed. Regular monthly meetings were held to establish and ensure a shared understanding of initial codes.

Larger team meetings, involving all authors, were conducted to collectively scrutinize and further refine emerging patterns in the qualitative data, ultimately confirming the identified themes and subthemes. Throughout the analysis process, written memos and meeting minutes were maintained to document our approach and decisions. Adhering to research and reporting standards, we followed the Standards for Reporting Qualitative Research outlined by O’Brien et al [31] when reporting this study.

### Data Integration

Integration occurred at 2 points in this study. First, the quantitative findings were used to inform the qualitative interview guide. Following an independent analysis of all qualitative and quantitative data, the data were integrated using a joint display as an analysis tool. During this analysis, qualitative data were used to explain and corroborate quantitative findings [32]. Quantitative findings were compared to qualitative themes to examine similarities and differences. Through this methodology, we were able to develop interpretations regarding educators’ perceptions and experiences.

### Ethical Considerations

We obtained approval from our local Conjoint Health Research Ethics Board (REB22-0748) to carry out this study. Educators were offered the opportunity to join the study voluntarily, with the assurance that their involvement in the survey would remain anonymous and would not affect their university employment status or career advancement. Completion and submission of the online surveys implied consent. Before participating in interviews, all respondents gave informed verbal consent. Interviews were administered by a graduate student who had no prior supervisory relationship with the participants. To protect participant anonymity, distinct identifiers were assigned to each participant, and the data were aggregated accordingly. No compensation was provided to participants for participating in this study.

### Rigor

We used several techniques to ensure the rigor of our study. Regular team meetings provided opportunities for debriefing, introspection, and deliberate questioning of our interpretations, as suggested by Morse [33]. We maintained a comprehensive audit trail that included codebooks, meeting minutes, and shared files to document all study-related decisions, following the guidelines proposed by Carnevale [34]. While 2 researchers were responsible for coding all qualitative data, the broader research team assessed and deliberated on decisions related to themes and subthemes. We revisited the raw survey and interview data to further validate our findings and ensure that they authentically represented the voices of the educator participants.

## Results

### Participant Demographics

A total of 82 educators started the survey, and 72 (88%) completed the entire survey. The 10 (12%) participants who did not complete the entire survey completed up to the final 5 survey items. We included all responses provided by participants in our final analysis as they yielded valuable insights and contributed to our overall study findings. Of the 82 survey participants, 19 (23%) agreed to be contacted for a follow-up interview of which 8 (10%) responded and completed an interview. Table 1 provides participant demographics for the survey and interviews.

**Table 1:** Participant demographics.

Demographic and demographic subcategory		Survey (n=82), n (%)	Interview (n=8), n (%)
**Age (y)**			
	<39	11 (13)	0 (0)
	40-49	25 (30)	1 (13)
	50-59	29 (35)	3 (38)
	>60	16 (20)	4 (50)
	No response	1 (1)	0 (0)
**Gender**			
	Men	18 (22)	2 (25)
	Women	58 (71)	6 (75)
	Gender diverse^a^	6 (7)	0 (0)
**Faculty**			
	Education	21 (26)	3 (38)
	Medicine	34 (41)	3 (38)
	Nursing	16 (20)	2 (25)
	Social work	7 (9)	0 (0)
	Other (joint appointments)	4 (5)	0 (0)
**Experience^b^**			
	Beginner	35 (43)	3 (38)
	Intermediate	24 (29)	2 (25)
	Expert	23 (28)	3 (38)

^a^Gender diverse included gender fluid, nonbinary, queer, and individuals who prefer not to disclose. Some categories were collapsed due to the need to maintain anonymity, particularly with small numbers in particular subcategories.

^b^Beginner=taught <4 online courses; intermediate=taught 5-7 online courses; expert=taught ≥8 online courses.

### Quantitative Results

#### Overview

**Figure 1:**
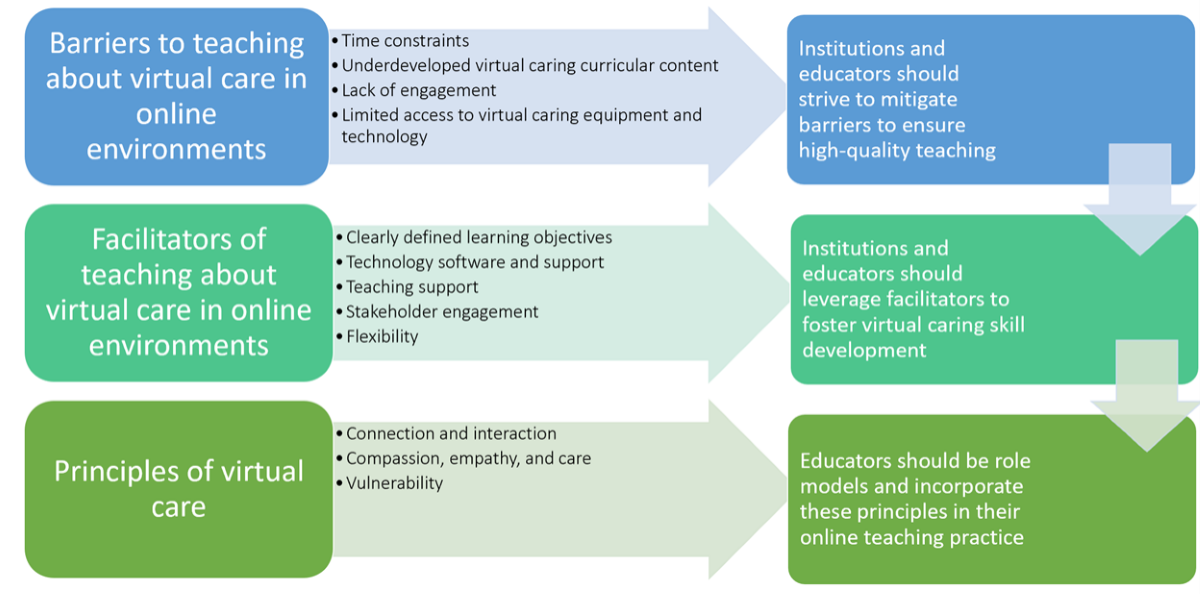
Overview of themes, subthemes, and implications.

Educator survey respondents (n=82) indicated that a variety of online instructional methods were used to help students develop virtual caring skills in a *select all that apply* survey item (Figure 1). The most frequently reported online instructional methods included using reflections on learning (50/82, 61%), online modules (35/82, 43%), and online discussion boards (49/82, 60%). Educators reported using demonstrations of remote care (23/82, 28%) and consultation with clients (21/82, 26%). Respondents that used the option of *other* (7/82, 9%) described using verbal check-ins, synchronous meetings, simulations, social media, and flipped classrooms. Some respondents indicated that they have not used any online instructional methods to develop virtual caring skills (17/82, 21%).

#### Satisfaction With Online Teaching and Learning Strategies

Survey participants (n=80) reported their level of satisfaction with online teaching and learning strategies, with 71 (89%) participants indicating that they were either satisfied or somewhat satisfied with the approaches used in their classrooms. However, a notable proportion, approximately 11% (9/80) of the participants, reported dissatisfaction.

#### Likelihood of Using Online Teaching and Learning Technologies

Among educators who responded to the question (n=70) about the likelihood of using online teaching and learning technologies to support students in developing virtual caring skills in the future, 53 (76%) indicated that they were very likely or somewhat likely to engage in this modality. Conversely, 17 (24%) educators responded that they were not likely to use online teaching and learning for the development of virtual caring skills in the future.

We conducted 1-way ANOVA tests to explore potential differences in satisfaction and likelihood to use technology scores among groups based on gender, age, faculty, years of experience in current position, or experience with online teaching and learning technologies. Table 2 summarizes the ANOVA test results.

In the survey, an expert was defined as an educator who had designed and taught ≥8 classes. There was a statistically significant difference between educators’ level of experience teaching online and their satisfaction with online teaching and learning technologies (*F*_2,77_=11.465; *P*<.001), with a large effect size (η^2^=0.23) [28]. A Bonferroni post hoc analysis demonstrated that educators with expert experience with teaching using technology reported significantly higher satisfaction (mean 2.82, SD 0.39) compared to those at beginner (mean 2.06, SD 0.69) or intermediate levels (mean 2.21, SD 0.59). No statistically significant difference was found between those at beginner and intermediate levels. A statistically significant difference was found between educators’ faculties (departments) and their satisfaction with online teaching and learning technologies (*F*_4,75_=5.119; *P*=.001), with a large effect size (η^2^=0.21). A Bonferroni post hoc analysis found that educators from the faculty of education (mean 2.75, SD 0.44) rated their satisfaction with online teaching and learning technologies significantly higher than faculty from medicine (mean 2.12, SD 0.64) or nursing (mean 2.07, SD 0.70). There were no statistically significant differences found between social work and the remaining faculties. Notably, all other comparisons via 1-way ANOVA tests yielded no statistically significant results.

Through Levene tests, two 1-way ANOVA test pairings were found to have unequal variances: (1) faculties and likelihood of using online teaching and learning technology and (2) years of experience and likelihood to use online teaching and learning technology. The Kruskal-Wallis *H* test, a nonparametric equivalent, was used to examine those relationships. A Kruskal-Wallis *H* test demonstrated that there was a statistically significant difference in the likelihood of using online teaching and learning technologies and the different faculties (*H*_4_=13.44; *P*=.009), with a mean rank likelihood of 52.0 for the faculty of social work, 43.2 for the faculty of nursing, 36.8 for the faculty of education, 34.6 for other faculties, and 27.0 for the faculty of medicine. A pairwise comparison revealed that educators from the faculty of social work had a significantly higher likelihood of using online teaching and learning technologies than the faculty of medicine when considering the Bonferroni correction for multiple tests, *P*=.01. This was the only significant relationship found in the pairwise comparison after applying the Bonferroni correction. Kruskal-Wallis *H* test demonstrated that there was no statistically significant difference in the likelihood of using online teaching and learning technologies and years of experience (*H*_5_=3.956; *P*=.56).

**Table 2:** ANOVA test results.

Variable comparison		Descriptive statistics		ANOVA					
		Participants, n (%)	Mean (SD)	*F* test (*df*)		η^2^		*P* value	
**Gender and satisfaction (n=80)**					1.38 (2, 77)		0.04		.26
	Men	18 (23)	2.11 (0.68)						
	Women	56 (70)	2.39 (0.65)						
	Gender diverse	6 (8)	2.17 (0.75)						
**Age (y) and satisfaction (n=80)**					0.31 (3, 74)		0.01		.82
	0-39	10 (13)	2.40 (0.52)						
	40-49	25 (31)	2.24 (0.66)						
	50-59	29 (36)	2.28 (0.80)						
	>60	14 (18)	2.43 (0.51)						
**Faculty and satisfaction (n=80)**					5.12 (4, 75)		0.21		.001
	Education	20 (25)	2.75 (0.44)						
	Medicine	34 (43)	2.13 (0.64)						
	Nursing	15 (19)	2.07 (0.70)						
	Social work	7 (9)	2.71 (0.49)						
	Other	4 (5)	2.00 (0.82)						
**Experience (y) and satisfaction (n=80)**					0.99 (5, 74)		0.06		.43
	<1	5 (6)	2.20 (0.84)						
	1-5	28 (35)	2.39 (0.63)						
	6-10	21 (26)	2.43 (0.68)						
	11-15	12 (15)	2.33 (0.65)						
	16-20	5 (6)	1.80 (0.84)						
	>20	9 (11)	2.11 (0.60)						
**Online experience and satisfaction (n=80)**					11.46 (2, 77)		0.23		<.001
	Beginner	34 (42)	2.06 (0.69)						
	Intermediate	24 (30)	2.21 (0.59)						
	Expert	22 (28)	2.82 (0.39)						
**Gender and likelihood to use (n=70)**					1.68 (2, 67)		0.05		.20
	Men	14 (20)	2.07 (0.92)						
	Women	52 (74)	2.38 (0.80)						
	Gender diverse	4 (6)	1.75 (0.96)						
**Age (y) and likelihood to use (n=70)**					0.28 (3, 36)		0.01		.84
	0-39	6 (9)	2.50 (0.55)						
	40-49	22 (31)	2.18 (0.91)						
	50-59	26 (37)	2.35 (0.89)						
	>60	14 (20)	2.29 (0.73)						
**Online experience and likelihood to use (n=70)**					1.92 (2, 67)		0.05		.16
	Beginner	27 (39)	2.11 (0.89)						
	Intermediate	22 (31)	2.23 (0.81)						
	Expert	21 (30)	2.57 (0.75)						

### Qualitative Findings

#### Overview

Figure 1 offers a summary of 3 overarching themes and their associated 12 subthemes, which were identified when analyzing the qualitative data. It also highlights potential recommendations for supporting online teaching to enhance the development of virtual caring skills. The subsequent sections delve deeper into the exploration of these findings.

#### Barriers to Teaching About Virtual Care in Online Environments

Educators identified several barriers that were encountered for online teaching and learning related to the development of virtual caring skills, including time constraints, underdeveloped virtual caring curricular content, a lack of engagement, and limited access to virtual caring equipment and technology. Despite these barriers, participants often highlighted their adaptability in addressing the needs of their students and teaching contexts.

#### Time Constraints

Participants reported time constraints as a concern, citing challenges such as the increased duration of virtual interactivity and the need to adapt clinical experiences for online platforms. Educators discussed time constraints as a limiting factor for teaching duration and described the need for adjustments. One participant shared the following:

Another barrier is always time, right?...For it not to be just text heavy and kind of interactive, you need time, and you don’t necessarily have that.

P6, interview, education educator

In some cases, the concern expressed was for students who could not be online for extended durations:

Clinical online, it’s eight hours online. We made a decision that was too much for the students to be online.

P1, interview, nursing educator

In addition to teaching time constraints, educators noted that additional time was required when offering experiential learning in practice:

Timing was always an issue. It seemed to take longer to do virtual appointments than in person.

P8, interview, medicine educator

The shift to virtual teaching and learning spaces prompted educators to be mindful of time constraints and their impacts. Despite these challenges, all participants adjusted to better cater to the needs of their students and those they would be caring for in practice.

#### Underdeveloped Virtual Caring Curricular Content

Educators reported difficulties in identifying key content related to virtual caring. As noted by a nursing educator, virtual caring content was often missing from the curriculum due to curricular overload:

Actually, I would say one of the things that I feel that is missing from the clinical practice for [this context] is the education part. We do a little bit of in the clinical, but to do the total education...we have to do it that way because there’s no time to include absolutely everything.

P1, interview, nursing educator

Virtual caring skills and competencies were often considered a specialized practice and were therefore not traditionally incorporated into more generalist-focused curricula. However, the onset of the COVID-19 pandemic made virtual care a crucial competency for many caring professions. An educator from the faculty of education noted how students tried to balance the unknown of virtual care expectations with how they may be expected to practice virtually when they graduated:

...but [students] don’t necessarily know what they’re getting into because you’re asking them to look at an area that is somebody else’s whole specialization, and yet we expect all teachers to know this information.

P3, interview, education educator

These responses demonstrated the challenges educators faced due to the lack of virtual caring curricular content, potentially negatively impacting students’ ability to provide virtual care in their future practice.

#### Lack of Engagement

Educators expressed concerns regarding the lack of student engagement they encountered in online settings when teaching about virtual care. They cited interruptions, decisions about cameras being on or off, and struggles connecting with students and colleagues as factors negatively impacting student engagement levels. The online environment posed a complex challenge, with frequent connectivity issues and interruptions. One interview participant noted the following:

We had cats and dogs. We had children interrupting...every time a student would come in [to the Zoom call], the doorbell [chime] would ring and [their] dog would go berserk. [They’d] be constantly shutting mute on, and then have to do something about that dog. So, [they]’d disappear from the screen and then come back.

P1, interview, nursing educator]

The debate over whether students should have their cameras switched on or switched off during virtual learning was raised, particularly in terms of establishing a sense of presence:

To providing an ethic of care is the cameras on cameras off issue...the preference for students to have their cameras off makes for a very difficult teaching environment...I can’t see your face; I can’t see your reaction.

P3, interview, education educator

Beyond visible student presence in a virtual class, educators also raised a concern about how virtual caring challenged their own engagement and sensory perceptions:

I can’t sense what’s going on for them...You can’t feel the energy in the room, right? You can’t see body language. You can’t see nonverbal communication...These are professions where we rely on all of our senses. And in a virtual environment, they’re not all there.

P6, interview, education educator

One survey respondent identified the following:

I can’t see faces or check in with people who might show signs of confusion the same way I can in person. You can’t “read the room” online.

P23, survey, medical educator

#### Limited Access to Virtual Caring Equipment and Technology

Educators expressed concerns about the limited access to virtual caring equipment and technology, which had a detrimental impact on interactivity. For one educator in medicine, the lack of equipment was an ongoing challenge:

I would say that the interaction suffered. We struggled with not having enough private computer space in the hospital. We struggled with not having cameras for the learners, and microphones, and that went on for quite a while.

P8, interview, medicine educator

For a nursing educator, the lack of student internet access was a challenge in teaching virtual care and creating environments for students to practice their virtual caring skills:

And there was one student who had to do [Zoom] on [their] cell phone, and she was using her minutes on her phone. It was getting too expensive. It was so much better if [they] just didn’t use [their] cell phone...there were other students, their internet would go down.

P1, interview, nursing educator

Teaching and providing virtual care in rural and remote areas brought attention to the privilege of internet access and resources as well as the challenges faced by clients:

There are limitations in some of the other countries about their access to Wi-Fi…many people do not have access to Wi-Fi at home. Therefore, the scheduling is important. I think many centers then also have interruptions of their Wi-Fi and are constantly on and off, on and off, and that creates some problems for them. And finally, there’s a few centers that the reason for that happening is that they lose electricity.

P5, interview, medicine educator

Remote learning in rural areas, it all depends on bandwidth...At the beginning, I didn’t realize the reason why people weren’t turning their cameras on...Tech is always a barrier...whether it’s bandwidth, whether it’s Zoom not working, whether our own internet.

P6, interview, education educator

The challenges, such as lack of equipment and poor internet accessibility, directly impacted educators’ ability to teach students virtual caring skills and competencies. These considerations can also be challenges in working with experts or patients outside of the virtual classroom.

#### Facilitators to Teaching About Virtual Care in Online Environments

Educators identified several facilitators for online teaching and learning related to virtual caring skills. These facilitators included well-defined learning objectives, supportive technology software and assistance, effective teaching support, active stakeholder engagement, and a commitment to flexibility.

#### Clearly Defined Learning Objectives

Educators brought up their awareness of key graduate expectations, competencies, and learning objectives in both the open-ended survey questions and interview responses. Some educators were challenged in aligning new virtual contexts with previously defined learning competencies:

I’ve had to reconsider how my own caring is conveyed and recognized in different circumstances. I’ve also begun to theorize about how caring is connected to key graduate learning expectations and competencies.

P66, survey, education educator

Other participants, like this one from medicine, asserted that the learning objectives should remain consistent despite the shift to online learning:

I don’t think we’ve changed the learning objectives. I think that they remain relatively constant, it’s how you achieve them. And with the remote learning, the remote learning has allowed the interaction, but it’s the interaction I think that’s more important than the virtual way of doing things.

P5, interview, medicine educator

Other educators spoke about how the processes of learning caring competencies might not change in virtual contexts, but students may struggle to see the value of acquiring virtual caring skills:

If they [students] don’t care about something, it doesn’t become part of a learning repertoire. Then what you have to then wonder if you’re just covering material for the sake of covering...it’s not enough that I care about the ideas, I need to get them to care about the ideas as well.

P4, interview, education educator

Despite the various viewpoints on how learning objectives were achieved, there was consistent support across the participants for the development and use of clear learning objectives related to virtual care. Particularly for participants who had relied previously on in-person assessments of learning objectives, there was an intentionality to focus and be explicit on what the learning objective was and how virtual care considerations were necessary.

#### Technology Software and Support

Participants identified that possessing knowledge and intentionally using technology and virtual caring software could enhance the development of virtual caring skills. Others identified the benefits of providing orientation and skill development sessions to familiarize individuals with the use of technology:

Some of the [online] programs demanded a lot of interaction...so it started off with teaching people how to do things [in the online programs].

P5, interview, medicine educator

Furthermore, survey participants asserted that ongoing technical assistance was important to successfully integrate new technologies into the virtual caring curriculum. One survey participant commented on the positive advancement of technology and its influence on education. They wrote, “The technology has come so far that teaching online is often equivalent to in person” (P51, survey, social work). Some of the examples of technology use included telehealth, podcasting, video creation, Zoom, and virtual simulations.

In one example with clinical practice, learners were actively engaged with a particular client population online. Students were tasked with using technology and software to interact with the client. Experimenting with the various features of the technology provided an additional way for students to learn new ways to establish connections with clients:

They hear the [diagnosis], and they’re like, oh, they can’t do anything. But they were having fun with the little apps that turn your hair green, or give you bunny ears, and stuff like that. So, they’re going through and playing with all that kind of stuff. I don't even know where half that stuff is or how they find it. But it's hilarious and it's fun to watch. So, it becomes a medium and a tool kind of thing.

P7, interview, nursing educator

It is important for students to gain a clear understanding of how to use virtual caring technologies efficiently and effectively to make meaningful caring connections with clients. The perspective from both survey and interview participants reinforced that having access to the tools was important, with support and familiarity requiring time and resources for tool use competency.

#### Teaching Support

Educators identified that various teaching supports were necessary for fostering initial self-awareness and skill development when teaching in virtual settings. Ongoing development and the exchange of best practices helped build and sustain confidence and competence in using virtual caring technology. Many educators turned to others for teaching support, including teaching and learning departments and teams, or external networks to help support their personal learning needs. Others found teaching support from within their own faculty and professional organizations:

My colleagues and my own field professional organization was better in terms of teaching strategies or things to do within a lesson.

P6, interview, education educator

Overall, educators were motivated to seek out ways to enhance their teaching practice of virtual caring skills.

#### Stakeholder Engagement

Study participants identified stakeholder support and engagement as important to virtual caring skill development. One survey respondent contributed that “online teaching is forcing me to get creative...I learned to rely more on facilitating students’ own motivation and initiative to seek community involvement” (P40, survey, medicine educator). Educators sought to encourage students to engage with clients in the community to help inform their virtual caring practices. Another survey participant indicated the importance of consulting various stakeholders, including students and educators, regarding their experiences with virtual caring technology by suggesting faculties should do the following:

[Engage in] consultation with students to understand their experiences as the end user/recipient of any technologies used for developing caring skills; [develop] a long-term vision/strategy for implementing, evaluating, and updating technology; [and link] technology use to program intent/pedagogy so that it makes sense to teachers/learners and is not just used for the sake of it.

P5, survey, nursing educator

Others highlighted the value of engaging with a range of stakeholders, including caregivers, clients, students, and instructors, in the virtual care setting:

We would invite the clients and the caregivers, or whoever was in the home to set up the screen and make sure that all of the controls were kind of off so that we could control it. And so as long as they could log in, we could get them into a breakout room. We would put the student in there with them. We would put a mentor from the [organization] in there with them. And then as instructors, we would go into each breakout room and just listen, make sure everything was okay, answer any questions, and then go to the next one and kind of wander through that way. And it worked really well.

P7, interview, nursing educator

Participants indicated that various stakeholders bring valuable and diverse perspectives to virtual caring experiences and harnessing these viewpoints can help facilitate more effective teaching and learning about virtual care.

#### Flexibility

Educators identified various ways that they chose to adjust, alter, change, or remain open to alternative ways of engaging in their practices for teaching, learning, and providing virtual care. The theme of flexibility emerged prominently in the survey responses, with a focus on being flexible with students. One survey participant emphasized the importance of “just being open and available and allowing students to set the stage for how they want to show up and learn and to be open if they are finding the online approach to learning challenging” (P79, survey, social work educator). Another perspective on flexibility was that it “allowed for more flexible scheduling and allow(ed) me to reach international students easier” (P15, survey, medicine educator). The connection to students in conducting, developing, or framing the learning space was recognized as a key element in building the flexibility to permit learning that incorporated virtual learning skills. This flexibility contributed to a more dynamic and inclusive learning environment.

#### Principles of Virtual Care

In our analysis, we identified principles of virtual care that reflect what educators reported as important considerations to how they approached teaching and learning virtual caring skills. These principles include emphasis on connection and interaction; compassion, empathy, and care; and vulnerability.

#### Connection and Interaction

Educators identified how important connections and interactions were for teaching about and providing virtual care. This perspective was particularly present for a nursing educator who described how technical nursing skills were not as important as making personal connections with the clients, which is vital when providing virtual care:

[Students] felt that they were missing out on some of those skills, like IV starts because obviously we didn’t do that [in a virtual environment]. But no, those are not the most important skills in nursing. It’s the interaction. It’s the education...nursing is not all about skills.

P1, interview, nursing educator

Some educators were thoughtful in their approach to providing students with purposeful opportunities to develop connections with clients:

I would want to be in a different room, with my camera off, observing the whole encounter...be the fly on the wall...and then be able to deliver feedback after the appointment.

P8, interview, medicine educator

All educators identified through the interviews that personal connections and prioritizing interactions were desired, and even necessary, before skill development in virtual environments.

#### Compassion, Empathy, and Care

Educators shared how emotional labor and intentional considerations are required to design learning experiences around compassion, empathy, and care, particularly in virtual contexts. One survey respondent suggested that “students of any caring profession know they need emotional bravery and an ability to handle very difficult situations with empathy and calmness even when they do not feel that way” (P34, survey, social work educator). Participants also indicated they needed this emotional bravery to successfully implement online teaching and learning technologies to support students in developing virtual caring skills. Educators acknowledged the impact and challenges associated with emotional labor and considered their role as educators in addressing issues like compassion fatigue:

Emotional labor and compassion fatigue...because those aspects impact the degree to which somebody wants to try something new or continue a practice that used to work, that doesn’t seem to be working now.

P3, interview, education educator

One interview participant considered the impact of learning activities with a focus on social and emotional learning for individual well-being:

I also am a very active and dynamic facilitator, even online, so I use teaching strategies that I would use in the classroom and I get my students to actually get up and do things if I’m talking about a social emotional learning activity, something that’s for wellbeing, because taking care of yourself is as important as you know what you’re teaching, and you will impact the wellbeing of your own students or patients by the way you are as well. So, if I’m talking about just a simple social-emotional piece where it is maybe a five, four, three, two mindfulness activity, I do it with them.

P6, interview, education educator

Compassion, empathy, and care were viewed as important considerations in teaching, learning, and providing virtual care. These qualities could manifest authentically in a variety of ways, depending on the context of the teacher, learner, or client.

#### Vulnerability

The theme of care extended to include a focus on educator vulnerability and the willingness to embrace new approaches, recognizing that things might not always go as planned. However, this willingness by the educator required creating safer learning and caring spaces:

In caring skills and competencies, there’s a level of vulnerability there that you must have. And so, when you’re starting out with online courses, you need to build that caring atmosphere within your virtual online environment in a way that students feel safe.

If you have a course, you have the time, and you utilize facilitation methods that are similar to what you are expecting them to be able to do as well, then that’s helpful, right? I guess it comes back to that theory practice piece.

P6, interview, education educator

Another participant spoke about the need to break down barriers by creating relationships that push virtual caring efforts to meet clients’ needs:

They [clients] put up their own barriers, to be perfectly honest. Because if you want it, you’ll find a way to do it. But…If you have the goal in mind that, then all you need to do is figure out how to get there. It’s a lot easier…I mean, create relationships. Ask people if they want to try something. And don’t think you can’t do it just because nobody’s done it before...See if it works. Not everything works the first time. Well, I know that’s why this is important too, right? It’s like you evaluate and you figure out what works, what doesn’t work.

P7, interview, nursing educator

There was a shared sense among participants that without the educator’s sense of vulnerability and willingness to try something new and create intentional efforts toward connection through compassion and care, educational practice for virtual care would not be able to move forward.

## Discussion

### Principal Findings

In this sequential explanatory mixed methods study [24] we explored the experiences and perceptions of educators in caring professions as they navigated online teaching to facilitate the development of virtual caring skills and competencies among students. Educators identified both barriers and facilitators to engaging in this mode of teaching and learning as well as identified key principles underlying virtual caring.

Quantitative and qualitative data were integrated following individual analysis. The most common online instructional methods used to teach virtual caring skills were reflection, online modules, and online discussion boards. Only 26% (21/80) of the participants indicated that they provided experiential learning via consultation with clients on the quantitative survey. In qualitative interviews, participants discussed barriers to this educational modality, such as lack of time, indicating that providing virtual caring experiences could be less efficient than providing in-person clinical learning. Furthermore, 21% (17/80) of the educators indicated that they had not used online technology to teach virtual caring skills. This was reflected in the qualitative data when participants discussed the challenges of fitting more content into an already crowded curriculum. As virtual environments increase in the caring professions, it is important that virtual caring curriculum becomes a more permanent fixture within program curricula [35], rather than treated as a specialty consideration that can be included if time permits. This highlights the attention for program-level considerations for technological literacy and use development. It is not enough for educators to be able to use the technology effectively and use tools in one course; instead, there is a need to identify opportunities across a program to support the learning and development of digital literacy and technology-use competencies.

Educators had varying levels of satisfaction with their online teaching and learning strategies to enhance virtual caring skills. Less than half of the participants (34/80, 43%) indicated that they were satisfied with their online teaching and learning strategies, with other educators indicating that they were either somewhat satisfied or not satisfied. Through the qualitative survey and interview data, educators expressed frustration regarding the lack of engagement or connection with their students, which created difficult teaching environments. Educators also expressed concern regarding students’ access to technology devices and reliable internet. Bolster et al [35] expanded this idea when discussing that clinical patients that might have limited access to virtual caring technologies or may lack digital literacy. In this study, the challenges discussed by educators may have influenced their overall satisfaction with their ability to execute effective teaching and learning strategies. In the survey qualitative responses, those that were “satisfied” (34/80, 43%) often cited reasons such as the smooth functioning of technology and active student engagement. Educator and student interactions with technology appear to be influential to educators’ satisfaction with the teaching experience. Leaders from across the United States emphasized the importance of optimizing the logistics of technology when they met for a symposium titled Crossing the Virtual Chasm: Rethinking Curriculum, Competency, and Culture in the Virtual Care Era [35]. They reported that the need to optimize logistics, including providing equitable technology access and user software training, was one of the levers that can improve virtual care education [35].

Although educators’ likelihood to use online teaching and learning technology was mixed in quantitative surveys, there was notable support to develop learning objectives to enhance virtual caring skills. Educators discussed facilitators that could enhance the teaching and learning of virtual caring skills in interviews. Survey respondents who identified as very likely to use online teaching and learning technologies (37/70, 53%) indicated via qualitative responses that teaching support through professional development, ongoing technology assistance, and student engagement was essential to support students in developing virtual caring skills. Addressing challenges that arise while teaching and learning virtual caring skills in an online environment can be beneficial to student outcomes and educators’ satisfaction and increase their likelihood to use such technologies. Although higher education institutions are working to keep up with evolving technologies, specialized attention will be required in the virtual caring education context [35].

Surveys and interviews were undertaken with educators across caring professions, including education, medicine, nursing, and social work, within a research-intensive educational institution in western Canada. Quantitative analysis revealed interesting insights into educators’ satisfaction with online teaching and learning strategies and their likelihood to use online teaching and learning technologies. Overall, educators were somewhat satisfied with the online teaching and learning strategies they were using in their classrooms. Furthermore, they felt that they were somewhat likely to use online teaching and learning technology to support student learning of virtual caring skills. Through inferential analysis, we found that educators with experience designing and teaching ≥8 classes (considered expert level) had statistically greater satisfaction with the teaching and learning techniques they used in online learning environments. This finding indicates that educators could benefit from more experience in online teaching. This is congruent with the findings reported by Rhode et al [7], indicating that educators with more experience teaching in online environments had more positive attitudes toward online teaching and learning.

In addition, we found that educators from the faculty of education reported significantly higher satisfaction levels in teaching virtual care in an online modality compared to their counterparts in medicine or nursing. This may be largely due to the longer history that education faculties may have had in providing instruction in an online environment. This finding highlights the importance of offering additional support and professional development to educators in traditionally in-person programs, enabling them to effectively meet the needs of an increasingly online student population. In an integrative review, Cutri and Mena [36] discuss the cultural and structural challenges of traditionally in-person educators transitioning to online teaching and learning, including the workload required and readiness to transition to the online environment. Considering these challenges, academic institutions should consider implementing robust professional development programs to better support faculty engaging in online teaching and learning, ensuring optimal support for students learning virtual caring skills.

Educators identified several barriers to online teaching and learning related to the development of virtual caring skills, including time constraints, underdeveloped virtual caring curricular content, lack of engagement, and limited access to virtual caring equipment and technology. Time constraints may pose a significant challenge for educators as they strive to cover comprehensive content within limited time frames. Furthermore, educators may struggle to find room for virtual caring skills within their current curriculum, recognizing that to include additional content, other content will have to be reduced or eliminated. The underdeveloped nature of virtual caring curricular content may result in teaching and learning practices that lack the depth and breadth required to adequately prepare students for the nuances of virtual care. A notable barrier to teaching virtual caring skills in online environments, seen in this study and the literature, is the struggle to maintain student engagement, as online settings often hinder active participation and interaction. Students are more likely to be engaged when they have active learning opportunities, a positive learning climate, and meaningful interaction with faculty and peers [37]. Furthermore, limited access to virtual caring equipment and technology has exacerbated the challenge of teaching online [38] and hindered caring professionals’ practical application of virtual care concepts [39]. Addressing these barriers is crucial to ensuring a robust and effective virtual care education within online learning environments.

Educators in this study identified several facilitators of online teaching and learning related to virtual caring skills, such as clearly defined learning objectives, technology software and support, teaching support, stakeholder engagement, and flexibility. Clear and well-defined learning objectives play a pivotal role in ensuring quality education, providing a road map for both educators and students to navigate curriculum with clarity and purpose. Adequate technology software and support are essential facilitators, enabling seamless integration of virtual caring skills into the online environment. Teaching support, including resources, training, and guidance, enhances educators’ ability to effectively convey virtual caring concepts. In a grounded theory study, Shepherd et al [40] explored medical faculty and learner experiences regarding the learning of virtual caring skills during the COVID-19 pandemic. Despite medical faculty recognizing how virtual care can benefit patients, they were reluctant to continue to teach in virtual clinics, due to barriers at the individual, institutional, and systemic levels, citing challenging technology platforms and a lack of professional development as 2 of the limitations [40]. Stakeholder engagement, involving collaboration with health care professionals, institutions, and communities, may foster a more holistic approach to virtual care education. In addition, flexibility in instructional methods and assessment allows for adaptive learning experiences, catering to diverse student needs and optimizing the acquisition of virtual caring skills in an online setting.

Educators identified connection and interaction; compassion, empathy, and care; and vulnerability as key considerations when developing online teaching and learning experiences to support students in developing virtual caring skills. Fostering meaningful connections and interactions within the virtual learning space is essential for educators to create engaging and supportive learning environments. Encouraging compassionate and empathetic attitudes is fundamental, as these qualities are at the core of effective virtual care. Our findings mirrored the assertion by Bolster et al [35] that connection in virtual care is an essential component of “webside manner,” indicating the importance of rapport building through technology. Integrating opportunities for students to understand and express vulnerability is equally important, as it promotes authenticity and a deeper understanding of the human aspect of health care. By prioritizing these elements, online educational experiences can transcend physical barriers, providing a rich and holistic foundation for students to develop the interpersonal skills necessary for effective virtual caring [16,41-44].

This study is part of a larger multistudy research project intended to provide a framework for virtual caring skill development in higher education. This study explores the educator’s perspectives, while another study explores the student’s perspectives. The final integrated findings will inform a framework to guide educators from varied professions as they develop virtual caring curricula. By gaining educator and student perspectives, we aim to provide a comprehensive view of core principles, competencies, teaching methods, facilitators, and barriers to teaching and learning virtual caring skills.

### Strengths and Limitations

Our sequential explanatory mixed methods study provided a thorough examination of caring profession educators’ perceptions of virtual caring skill development within a specific educational institution. The inclusion of participants from various caring professions offered diverse perspectives, enhancing the study’s comprehensiveness. By incorporating surveys and interviews, the research amalgamated quantitative and qualitative data, enabling a more profound insight into educators’ experiences and perspectives in online teaching related to virtual care. However, it is essential to acknowledge the study’s limitations, warranting caution in interpreting the findings. The focus on a singular institution may limit the generalizability of these findings to broader contexts. Furthermore, the participant pool from a single institution may lack diversity, potentially affecting the external validity and transferability of findings to a more varied population. Despite these constraints, this study lays the groundwork for exploring virtual caring skill development, inspiring further research, and offering potential insights for enhancing the delivery of virtual care in educational settings.

### Conclusions

Educators in caring professions require specialized knowledge and skills to effectively teach and support students in developing virtual caring skills and competencies. Our study highlights the barriers, facilitators, and principles of teaching virtual caring skills online. As we contribute to the growing body of educational research on virtual caring skills, we share insights from caring profession educators. Future research should continue to explore how educators in more traditionally in-person teaching and learning can be supported to meet modern-day needs. In addition, more evidence is needed to explore effective teaching and learning strategies to teach virtual caring skills in a variety of contexts. Our findings offer practical strategies to enhance teaching and learning within educational programs for caring professions.

## References

[1] Bogo M (2015). Field education for clinical social work practice: best practices and contemporary challenges. Clin Soc Work J.

[2] Leading work-integrated learning in Canada. Future Skills Center.

[3] Dewart G, Corcoran L, Thirsk L, Petrovic K (2020). Nursing education in a pandemic: academic challenges in response to COVID-19. Nurse Educ Today.

[4] Roskvist R, Eggleton K, Goodyear-Smith F (2020). Provision of e-learning programmes to replace undergraduate medical students' clinical general practice attachments during COVID-19 stand-down. Educ Prim Care.

[5] Van Nuland S, Mandzuk D, Tucker Petrick K, Cooper T (2020). COVID-19 and its effects on teacher education in Ontario: a complex adaptive systems perspective. J Educ Teach.

[6] Dede C, Jass Ketelhut D, Whitehouse P, Breit L, McCloskey EM (2008). A research agenda for online teacher professional development. J Teach Educ.

[7] Rhode J, Richter S, Miller T (2017). Designing personalized online teaching professional development through self-assessment. TechTrends.

[8] Darling-Hammond L, Hyler ME (2020). Preparing educators for the time of COVID … and beyond. Eur J Teach Educ.

[9] Quezada RL, Talbot C, Quezada-Parker KB (2020). From bricks and mortar to remote teaching: a teacher education program‘s response to COVID-19. J Educ Teach.

[10] Cleland J, McKimm J, Fuller R, Taylor D, Janczukowicz J, Gibbs T (2020). Adapting to the impact of COVID-19: sharing stories, sharing practice. Med Teach.

[11] Ferri F, Grifoni P, Guzzo T (2020). Online learning and emergency remote teaching: opportunities and challenges in emergency situations. Societies.

[12] Kidd W, Murray J (2020). The COVID-19 pandemic and its effects on teacher education in England: how teacher educators moved practicum learning online. Eur J Teach Educ.

[13] Sepulveda-Escobar P, Morrison A (2020). Online teaching placement during the COVID-19 pandemic in Chile: challenges and opportunities. Eur J Teach Educ.

[14] Chittleborough G (2017). Learning how to teach chemistry with technology: pre-service teachers’ experiences with integrating technology into their learning and teaching. J Sci Teach Educ.

[15] Cantone RE, Palmer R, Dodson LG, Biagioli FE (2019). Insomnia telemedicine OSCE (TeleOSCE): a simulated standardized patient video-visit case for clerkship students. MedEdPORTAL.

[16] Lister M, Vaughn J, Brennan-Cook M, Molloy M, Kuszajewski M, Shaw RJ (2018). Telehealth and telenursing using simulation for pre-licensure USA students. Nurse Educ Pract.

[17] Love R, Carrington JM (2020). Introducing telehealth skills into the Doctor of Nursing practice curriculum. J Am Assoc Nurse Pract.

[18] O’Connor EA, Worman T (2018). Designing for interactivity, while scaffolding student entry, within immersive virtual reality environments. J Educ Technol Syst.

[19] Woodcock S, Sisco A, Eady M (2015). The learning experience: training teachers using online synchronous environments. J Educ Res Inst.

[20] Müller AM, Goh C, Lim LZ, Gao X (2021). COVID-19 emergency eLearning and beyond: experiences and perspectives of university educators. Education Sciences.

[21] Williamson B, Eynon R, Potter J (2020). Pandemic politics, pedagogies and practices: digital technologies and distance education during the coronavirus emergency. Learn Media Technol.

[22] Zhang W, Wang Y, Yang L, Wang C (2020). Suspending classes without stopping learning: China’s education emergency management policy in the COVID-19 outbreak. J Risk Financial Manag.

[23] Nowell L, Dhingra S, Carless-Kane S, McGuinness C, Paolucci A, Jacobsen M, Lorenzetti DL, Lorenzetti L, Oddone Paolucci E (2022). A systematic review of online education initiatives to develop students remote caring skills and practices. Med Educ Online.

[24] Creswell JW (2020). A Concise Introduction to Mixed Methods Research, 2nd Edition.

[25] Rattray J, Jones MC (2007). Essential elements of questionnaire design and development. J Clin Nurs.

[26] Topolovec-Vranic J, Natarajan K (2016). The use of social media in recruitment for medical research studies: a scoping review. J Med Internet Res.

[27] Yuan P, Bare MG, Johnson MO, Saberi P (2014). Using online social media for recruitment of human immunodeficiency virus-positive participants: a cross-sectional survey. J Med Internet Res.

[28] Polit DF, Beck CT (2013). Nursing Research: Generating And Assessing Evidence For Nursing Practice, 11th Edition.

[29] Braun V, Clarke V (2006). Using thematic analysis in psychology. Qual Res Psychol.

[30] Nowell LS, Norris JM, White DE, Moules NJ (2017). Thematic analysis. Int J Qual Methods.

[31] O'Brien BC, Harris IB, Beckman TJ, Reed DA, Cook DA (2014). Standards for reporting qualitative research: a synthesis of recommendations. Acad Med.

[32] Younas A, Durante A (2023). Decision tree for identifying pertinent integration procedures and joint displays in mixed methods research. J Adv Nurs.

[33] Morse JM (2015). Critical analysis of strategies for determining rigor in qualitative inquiry. Qual Health Res.

[34] Carnevale FA (2002). Authentic qualitative research and the quest for methodological rigour. Can J Nurs Res.

[35] Bolster MB, Chandra S, Demaerschalk BM, Esper CD, Genkins JZ, Hayden EM, Tan-McGrory A, Schwamm LH, Virtual CareMedical Educator Group (2022). Crossing the virtual chasm: practical considerations for rethinking curriculum, competency, and culture in the virtual care era. Acad Med.

[36] Cutri RM, Mena J (2020). A critical reconceptualization of faculty readiness for online teaching. Distance Educ.

[37] Cole AW, Lennon L, Weber Nl (2019). Student perceptions of online active learning practices and online learning climate predict online course engagement. Interact Learn Environ.

[38] Kuntz J, Manokore V (2022). “I did not sign up for this”: student experiences of the rapid shift from in-person to emergency virtual remote learning during the COVID pandemic. High Learn Res Commun.

[39] Ortega G, Rodriguez JA, Maurer LR, Witt EE, Perez N, Reich A, Bates DW (2020). Telemedicine, COVID-19, and disparities: policy implications. Health Policy Technol.

[40] Shepherd L, McConnell A, Watling C (2022). Good for patients but not learners? Exploring faculty and learner virtual care integration. Med Educ.

[41] Goldingay S, Boddy J (2016). Preparing social work graduates for digital practice: ethical pedagogies for effective learning. Aust Soc Work.

[42] Liu C, Scott KM, Lim RL, Taylor S, Calvo RA (2016). EQClinic: a platform for learning communication skills in clinical consultations. Med Educ Online.

[43] Pullen Jr RL, Silvers CA (2018). Helping students embrace HIT. Nurs Manage.

[44] Rutledge C, Hawkins EJ, Bordelon M, Gustin TS (2020). Telehealth education: an interprofessional online immersion experience in response to COVID-19. J Nurs Educ.

